# Mechanical rigidity of the Ortho-SUV frame compared to the Ilizarov frame in the correction of femoral deformity

**DOI:** 10.1007/s11751-015-0214-6

**Published:** 2015-02-26

**Authors:** Petr V. Skomoroshko, Victor A. Vilensky, Ahmed I. Hammouda, Matt D. A. Fletcher, Leonid N. Solomin

**Affiliations:** 1 Vreden Russian Research Institute of Traumatology and Orthopedics, 8 Baykova 8 Str., St. Petersburg, 195427 Russia; 2 Orthopedic Surgery Department, Al-Azhar University Hospitals, 10B Eltaka Street, El-Mabousin Buildings, 8th Area, Nasr City, Cairo, 11371 Egypt; 3 Department of Orthopaedics, Dawson Creek and District Hospital, 11100-13th Street, Dawson Creek, BC V1G 3W8 Canada; 4 St. Petersburg State University, 7-9 Universitetskaja Str., St. Petersburg, 199034 Russia

**Keywords:** Osteotomy, Fracture, Biomechanical stability, External fixator, Hexapod, Rigidity of osteosynthesis

## Abstract

The Ortho-SUV frame (OSF) is a novel hexapod circular external fixator which draws upon the innovation of the Ilizarov method and the advantages of hexapod construction in the three-dimensional control of bone segments. Stability of fixation is critical to the success or failure of an external circular fixator for fracture or osteotomy healing. In vitro biomechanical modelling study was performed comparing the stability of the OSF under load in both original form and after dynamisation to the Ilizarov fixator in all zones of the femur utilising optimal frame configuration. A superior performance of the OSF in terms of resistance to deforming forces in both original and dynamised forms over that of the original Ilizarov fixator was found. The OSF shows higher rigidity than the Ilizarov in the control of forces acting upon the femur. This suggests better stabilisation of femoral fractures and osteotomies and thus improved healing with a reduced incidence of instability-related bone segment deformity, non-union and delayed union.

## Introduction

The use of circular external fixation is reported extensively in the orthopaedic literature [1, 2]. The Ilizarov method has evolved to be used with a new generation of hexapod fixators which provide a number of benefits over the traditional design [3–5]. These have been employed with increasing frequency for the management of multiple pathologies [5].

The Ortho-SUV frame (OSF, Pitkar Orthotools, Pune, India) is a novel computer-aided hexapod fixator which addresses a number of deficits seen in other hexapod fixators. It has the advantage of a modular and changeable construction that can be customised to the limb segment more simply than other devices.

Stability of an external fixation device is critical. With insufficient stability, there is a risk of loss of position, excessive motion, failure of union or consolidation and pain. Conversely, with too much rigidity, the biologically desirable characteristics of stimulation through micromovement are diminished, with delayed consolidation and possible non-union [6, 7].

The amount of stability depends on both the particular type of pathology being addressed and the mechanical characteristics of the limb segment treated. Rigidity can be increased or decreased by varying the number and type of transosseous fixation elements but depends also on the particular characteristics of the struts or rods joining neighbouring rings [5, 8].

This study was designed to assess the degree of stability of the OSF in the femur in comparison with the traditional Ilizarov frame and to assess the decrease in rigidity when the frame is dynamised.

## Materials and methods

**Fig. 1:**
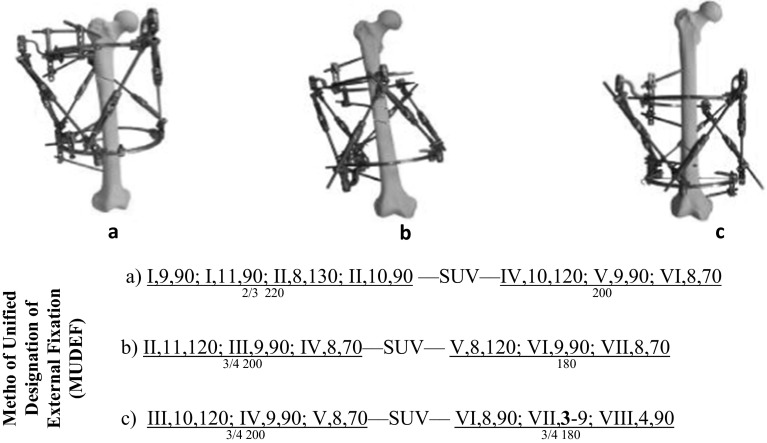
Optimum configuration and MUDEF of the OSF for the correction of deformities at the level of **a** proximal, **b** middle and **c** distal third of the right femur

Prior experiments on frame design for correction of proximal, middle and distal third femoral deformity have been studied in our department, and the optimal configuration was determined [9]. An intercalary ring distance of 150 mm was found to be the best for the maximum corrective potential of the frame. An optimal femoral frame configuration was designated according to the method for the unified designation of external fixation (MUDEF) assemblies [5, 10] and is shown in Fig. 1 for the right femur.

The MUDEF coordinates provide a reproducible classification of the insertion of frame elements thus: proximal ring elements, hexapod struts, distal ring elements. Within each element, the location of each pin is determined by the segmental level of the long bone (I proximal to VIII distal), the circumferential position of insertion (clock face, with 12 anterior, 3 medially, 6 posterior, 9 laterally) and the angle of insertion relative to the long axis (e.g. 90° perpendicular). The denominator describes the ring type and diameter (e.g. 3/4 ring with 200 mm diameter).

Hence, the MUDEF for Fig. 1a describes a proximal ring of 2/3 shape with a diameter of 220 mm, fixed by four pins: two at the most proximal epimetaphyseal zone, inserted at 90° to the long axis in positions 9 and 11 (direct lateral and anterior/lateral), and two further pins inserted in the proximal metaphyseal zone, the first just posterior of direct lateral inserted at 130° obliquity and the second just anterior of lateral at 90°. This proximal ring construct is joined by the OSF struts to the distal frame construct comprising a full ring of 200 mm diameter affixed with three further pins over three separate levels.

### The proximal third of the femur

In the proximal ring, strut number 1 is in position 12, strut number 3 in position 6, and strut number 5 in position 10. In the distal ring, strut number 2 is in position 3, strut number 4 in position 7, and strut number 6 between positions 10 and 11. Z-shaped plates are used to fix struts number 1 and number 5.

### The middle third of the femur

In the proximal ring, strut number 1 is in position 12, strut number 3 in position 5, and strut number 5 between positions 8 and 9. In the distal ring, strut number 2 is in position 3, strut number 4 between positions 6 and 7, and strut number 6 in position 11. Z-shaped plates are used to fix struts number 1 and number 5.

### The distal third of the femur

In the proximal ring, strut number 1 is in position 2, strut number 3 between positions 5 and 6, and strut number 5 in position 10. In the distal ring, strut number 2 is in position 4, strut number 4 in position 8, and strut number 6 in position 12. Z-shaped plates are used to fix struts number 1 and number 5.

 External fixator rigidity testing was carried out according to the “Method for Rigidity Testing of External Fixation Assemblies”, which provides a repeatable technique for comparison of rigidity between frames of differing design and construction by precisely specifying the configuration of the testing assembly for each plane of deformation, application of the frame within the testing apparatus, transducer placement and application of deforming forces to the frame construct, and the criteria for determining the rigidity parameters thus calculated [5, 10].

**Fig. 2:**
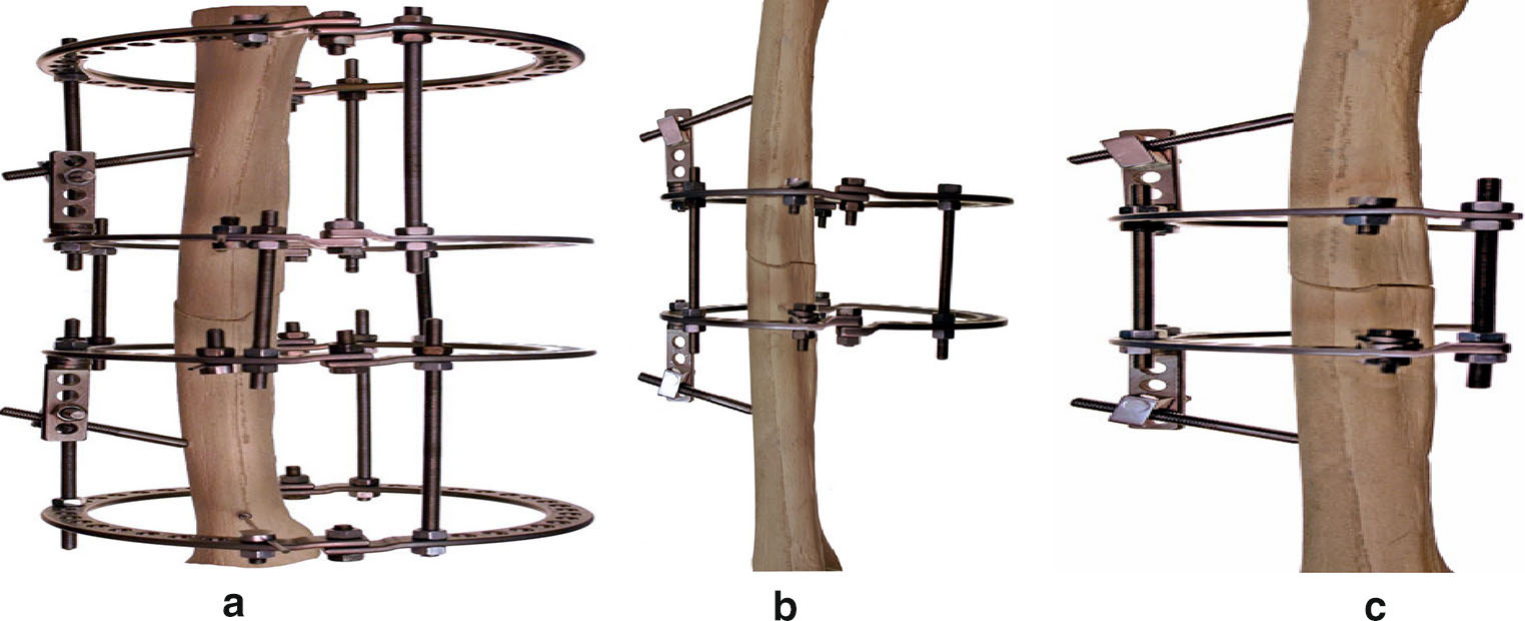
General scheme of frame dynamisation **a** basic frame assembly; **b** first stage: removal of the most proximal and distal rings; **c** second stage: partial removal of half of the rings

Rigidity of fixation using each respective frame was tested both in initial configuration and after modular transformation, known as dynamisation, which reduces frame rigidity to permit critical regenerate training as described by Ilizarov [1]. Dynamisation can be achieved by gradually decreasing the quantity of transosseous wires or pins, releasing tension from the wires, removing connecting rods between rings or unlocking struts, removing whole rings from a ring block, or releasing tension or compression from the system [5]. Dynamisation reduces pin-induced joint stiffness and increases patient tolerance due to reduction in the bulkiness of the frame (Fig. 2).

**Fig. 3:**
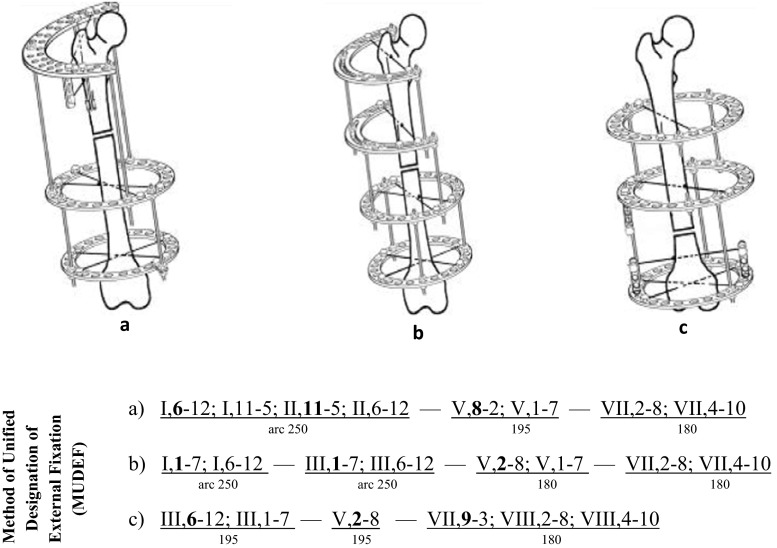
Ilizarov configuration and MUDEF for the **a** proximal, **b** middle, **c** distal thirds of a femur

Results obtained were compared with those observed with the conventional Ilizarov device (Figs. 2, 3).

**Fig. 4:**
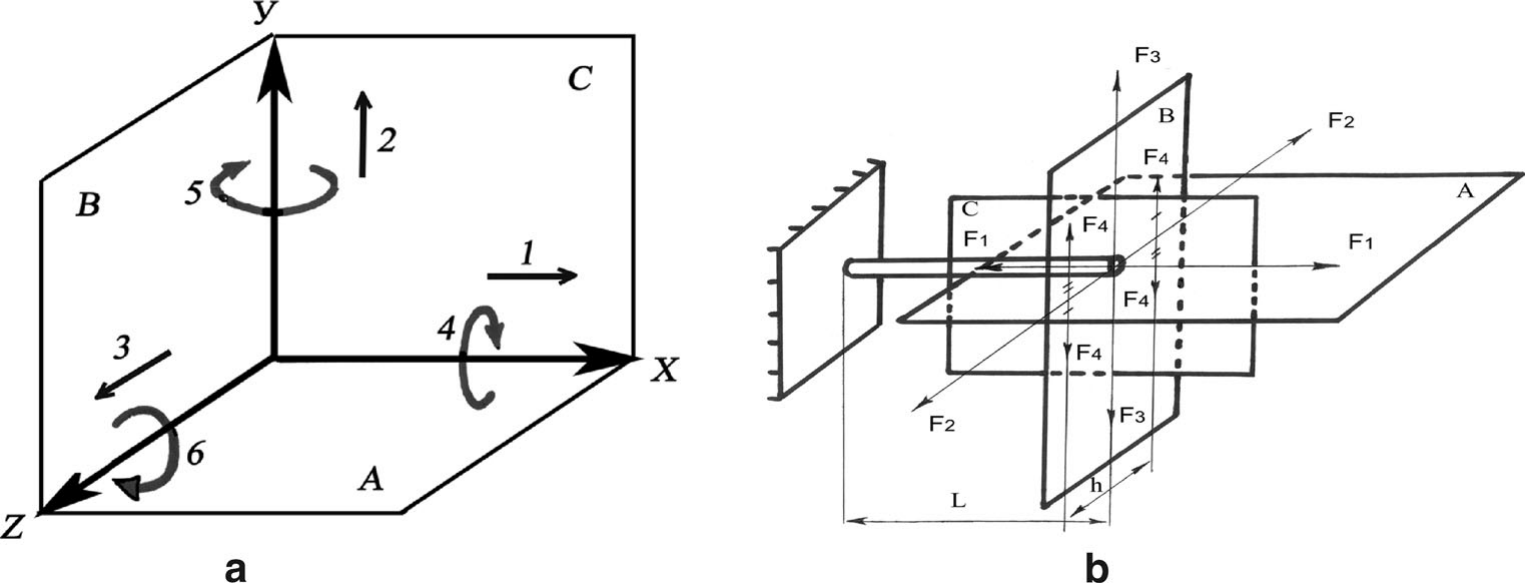
Schematic of standard displacing loads. **a** Possible displacement according to degrees of freedom, **b** loading scheme. *F*1 longitudinal distraction/compression force, *F*2 transverse abduction/adduction force, *F*3 transverse flexion/extension force, *F*4 rotational inward/outward force, *A* frontal plane, *B* transverse (horizontal) plane, *C* sagittal plane

All frames were assembled according to the “method for the unified designation of external fixation (MUDEF)”. This permits a replication of the experiments and verification of data. MUDEF provides a comprehensive system for the type and spatial orientation of wires and pins, order and direction of their placement, type of rings and the relationship between the rings [5, 8] (Figs. 3, 4).

Bone simulation within this study was performed with wooden rods, 30 mm in diameter and 500 mm in length. This has previously been described as providing the best approximation of bone in terms of mechanical characteristics and minimises the inaccuracy of other bone simulators; this allows standardisation of the testing not easily achievable with cadaveric material due to anthropomorphic variability [5].

The response to applied mechanical loads in six degrees of freedom was assessed for each frame construct (Fig. 4). Rigidity was determined by the ability of the fragments to resist displacement along the following parameters (Fig. 4):

1. F1—distraction and compression forces: longitudinal rigidity of the frame in response to distraction and compression.
2. F2—abduction and adduction forces: lateral rigidity of the module in the frontal plane.
3. F3—flexion and F3 extension forces: lateral rigidity of the module in the sagittal plane.
4. F4—medial and F4 lateral forces: the rotational rigidity of the module in response to medial and lateral displacement.

When displacement of the loaded bone simulator reached 1 mm or 1°, the load was deemed maximal. We compared the results obtained for each femoral level by the rigidity cofactor (*K*), which is the ratio of external loads to the linear and angular displacement. The higher this factor, the greater the rigidity of fixation of bone fragments. For example, the rigidity cofactors for distraction (*K*_distr_) and compression (*K*_compr_) were measured as follows:

$$K_{\text{distr}} = F1_{\text{distr}} /U_{\text{distr}}$$

and

$$K_{\text{compr}} = F1_{\text{compr}} /U_{\text{compr}}$$

whereby *U*_distr_ and *U*_compr_ describe fragment displacement in the axial direction by distraction and compression, respectively.

## Results

In each third of the femur, we determined the rigidity of the OSF in its initial configuration and after dynamisation. The results were then compared with those obtained for the rigidity of fixation using the Ilizarov frame [11]. Results are summarised for the proximal, middle and distal third femoral deformity, respectively (Tables 1, 2, 3).

**Table 1:** Frame rigidity in the proximal third of the femur

Plane and direction of displacing force	OSF	Dynamised OSF	Ilizarov frame^a^
Longitudinal rigidity, distraction, N/mm^b^	32 ± 1.0	26 ± 0.9	20
Longitudinal rigidity, compression, N/mm^b^	32 ± 1.0	26 ± 0.9	20
Frontal plane adduction, N/°^c^	50 ± 0.9	32 ± 0.8	1.3
Frontal plane abduction, N/°^c^	50 ± 0.9	32 ± 0.7	1.3
Sagittal plane flexion, N/°^c^	37 ± 0.8	28 ± 1.1	41
Sagittal plane extension, N/°^c^	37 ± 0.7	28 ± 1.1	41
Transversal plane, internal rotation, N/°^c^	27 ± 0.5	18 ± 0.7	18
Transversal plane, external rotation, N/°^c^	27 ± 0.5	18 ± 0.7	18

^a^[11]

^b^The unit for measuring the linear rigidity coefficient is Newton per millimetre (N/mm)

^c^The unit for measuring rigidity coefficient in other planes is Newton per degree (N/°)

**Table 2:** Frame rigidity in the middle third of the femur

Plane and direction of displacing force	OSF	Dynamised OSF	Ilizarov frame^a^
Longitudinal rigidity, distraction, N/mm^b^	43 ± 0.8	25 ± 0.7	18.6
Longitudinal rigidity, compression, N/mm^b^	43 ± 0.8	25 ± 0.7	18.6
Frontal plane adduction, N/°^c^	35 ± 0.8	21 ± 1.2	1.8
Frontal plane abduction, N/°^c^	35 ± 0.7	21 ± 1.2	1.8
Sagittal plane flexion, N/°^c^	29 ± 0.3	18 ± 0.7	27
Sagittal plane extension, N/°^c^	29 ± 0.3	18 ± 0.7	27
Transversal plane, internal rotation, N/°^c^	29 ± 0.4	21 ± 0.6	16
Transversal plane, external rotation, N/°^c^	29 ± 0.4	21 ± 0.5	16

^a^ [11]

^b^The unit for measuring the linear rigidity coefficient is Newton per millimetre (N/mm)

^c^The unit for measuring rigidity coefficient in other planes is Newton per degree (N/°)

**Table 3:** Frame rigidity in the distal third of the femur

Plane and direction of displacing force	OSF	Dynamised OSF	Ilizarov frame^a^
Longitudinal rigidity, distraction, N/mm^b^	35 ± 0.6	23.5 ± 1.0	28.5
Longitudinal rigidity, compression, N/mm^b^	35 ± 0.6	23.5 ± 1.0	28.5
Frontal plane adduction, N/°^c^	43 ± 0.8	16.5 ± 0.8	33
Frontal plane abduction, N/°^c^	43 ± 0.8	16.5 ± 0.8	33
Sagittal plane flexion, N/°^c^	18.5 ± 1.2	11 ± 1.6	16
Sagittal plane extension, N/°^c^	18.5 ± 1.2	11 ± 1.6	16
Transversal plane, internal rotation, N/°^c^	24 ± 0.7	18 ± 0.7	11.6
Transversal plane, external rotation, N/°^c^	24 ± 0.7	18 ± 0.7	11.6

^a^ [11]

^b^The unit for measuring the linear rigidity coefficient is Newton per millimetre (N/mm)

^c^The unit for measuring rigidity coefficient in other planes is Newton per degree (N/°)

Proximally, the OSF provides rigidity which exceeds that of the Ilizarov in the frontal plane by 38.5 times, in a transverse plane by 1.5 times, and in compression and distraction by 1.6 times. Stiffness of the OSF in the sagittal plane is similar to that of the Ilizarov. Dynamisation reduces the rigidity of the OSF by 1.2–1.6 times in different planes.

In the middle segment of the femur, the OSF provides rigidity of fixation which exceeds the rigidity of the Ilizarov in the frontal, sagittal, transverse and longitudinal planes by 19.3, 1.07, 1.8 and 2.3 times, respectively. Dynamisation reduces the rigidity of the OSF by 1.4–1.7 times in different planes.

Distally, the rigidity of the OSF is greater than that produced by the Ilizarov in the frontal and sagittal planes by 1.2 times. In the transverse and longitudinal planes, the rigidity of OSF exceeds that of Ilizarov by 2.07 and 1.2 times, respectively. Dynamisation reduces the rigidity of the OSF by 1.3–2.6 times in different planes.

## Discussion

Stability is affected by changing external fixator design and method of osseous fixation. For a meaningful and accurate comparison between different fixators and fixator constructs, standardised testing is necessary. MUDEF provides an accepted system of exact frame assembly for comparison [5, 8].

The rigidity provided by the OSF is greater than or equivalent to the Ilizarov in the femur when assembled in optimal configuration. Following dynamisation, the OSF approaches or just exceeds the rigidity of the Ilizarov in the majority of situations. These results support the use of the OSF in the management of femoral deformity correction where the increased ability to resist deforming loads due to muscular contraction or weight bearing can prove advantageous.

The mechanical characteristics of an external fixator influence the transmission of forces through an osteotomy or fracture site, and stability is key to controlling excursion and excessive motion [12]. Strain needs to be appropriately controlled; excess strain can inhibit bone formation and predispose to fibrous union. Conversely, too little strain, particularly with distraction, leads to atrophic non-union. Whilst greater rigidity has been suggested as conferring optimal results for bone union [12], the ideal external fixator rigidity remains unknown [12–14]. An initially rigid fixation followed by progressive dynamisation has been shown to be effective in achieving union and avoiding stress shielding [15].

The current literature does not compare and contrast hexapod and traditional Ilizarov frame rigidity. Fixator stability affects osteogenesis and so is critical [16]; optimal design for an external fixator is one that is rigid in torsion, bending and shear but allows for axial movement [17, 18]. Paley et al. [8] found the EBI and Orthofix (McKinney, TX, USA) monolateral external fixators to be more rigid than the Ilizarov frame, preventing axial motion at the osteotomy site. In contrast, greater loading of the bone ends was provided by the Ilizarov fixator but accompanied by the highest levels of shear [8]. In studying circular fixators, both Gasser et al. [19] and Podolsky and Chao [20] noticed that the nonlinearity of the load deformation curve exhibited by the Ilizarov frame in response to axial loading was not seen in the monolateral fixators. This nonlinear behaviour is reminiscent of the viscoelastic properties of biological structures and may be responsible for the promotion of fracture healing. The low frame rigidity seen at lesser loads allows more axial motion and is presumed to be useful for stimulation of callus formation. The higher frame rigidity seen at increased loads is thought to protect the healing bone from excessive motion. This property may explain how the Ilizarov frame has been able to promote osteogenesis where other frames have failed.

Some researchers have found that some hybrid and all-wire frames exhibit similar properties [21, 22]. Others have reported less ideal biomechanical characteristics for hybrid fixation in circular frames [23]. From our experience, we believe the hybrid fixation with the OSF to be more rigid, providing greater stability and as a result better healing.

The OSF, as tested in this study, is equal or better than the Ilizarov fixator in all zones in the femur and in all planes except in the proximal femoral third where the OSF has less rigidity in the sagittal plane. We believe this is due to the fact that the four-threaded Ilizarov rods are located substantially in the sagittal plane [11]. With the OSF, the struts lie in or near the frontal plane, which would explain the advantage of the OSF in frontal plane stiffness.

Dynamisation of an external fixator is important in regenerate training and consolidation of an osteotomy or fracture. One reported downside of the most frequently used hexapod, the Taylor spatial frame (TSF), is the lack of ease of achieving this. Controlled frame dynamisation with the TSF is not achievable easily due to the limited two-ring construct and interosseous transfixion [24]. Unlocking individual, alternate or all struts lead to an uncontrolled loss of stability in one or more planes [24]. This can be ameliorated by the use of non-standard modified shoulder bolts, which permit some motion between strut and ring, whilst preserving the overall configuration and relative stability, but this has not been proven. The OSF appears to exceed the mechanical characteristics of the Ilizarov fixator in terms of rigidity and allows controlled and safe dynamisation for desirable regenerate training without the risk of excessive and unwanted deformation. This may prove of clinical importance and will need to be confirmed in clinical studies.
